# Effective Aid for Hitting the Bull’s Eye: Comment on "It’s About the Idea Hitting the Bull’s Eye": How Aid Effectiveness Can Catalyse the Scale-up of Health Innovations"

**DOI:** 10.15171/ijhpm.2018.90

**Published:** 2018-10-02

**Authors:** Elvira Beracochea

**Affiliations:** Realizing Global Health Inc., Fairfax, VA, USA.

**Keywords:** Aid Effectiveness, Sustainability, Universal Coverage, Health Systems

## Abstract

This article studies how six key aid effectiveness principles for "Hitting the bull’s eye" can bring about the scale up of maternal and newborn health (MNH) interventions. These key principles are based on accepted international agreements such as the Paris Declaration on Aid Effectiveness. The results indicate that the six principles should be a guide for recipient countries to take ownership of the development process and work with donors to plan effective coordination structures. Countries that take ownership will be able to work with donors and implementers to not only test new interventions that address pressing challenges to deliver quality MNH care but also include the successful knowledge transfer and handover of these interventions, the effective integration of the new intervention as part of the country’s health system and a costed scale-up plan. The article could have been strengthened with clear and actionable recommendations for the three countries to improve their ownership of donor-funded assistance, but it showed that there is need to change how aid is delivered and that embracing and applying these principles will help countries take ownership of MNH programs and lead the dialog and effective scale up with those involved. The authors should be commended on taking the lead in the field of aid effectiveness, and encouraged to conduct quantitative and further qualitative measurements of the application of their findings in the three counties included in the study.

The article “It’s About the Idea Hitting the Bull’s Eye”: How Aid Effectiveness Can Catalyse the Scale-up of Health Innovations”^1^ studies how applying six key aid effectiveness principles can help “hit the bull’s eye” that is, scale up maternal and newborn health (MNH) interventions. The principles were selected based on the principles of the Paris Declaration of 2005^2^ and on other international agreements signed by the focus countries. They are well-defined in operational terms and include country ownership, alignment, harmonization, transparency and accountability, funding predictability, and participation of civil society organizations (CSOs).

The article shows evidence that the principles are generally accepted but not consistently applied in practice demonstrating the need for all involved to work differently if they are to “hit the bull’s eye.” The evidence comes from a qualitative study involving confidential interviews with 150 staff from recipient country governments, donor agencies and private donor foundations, and implementing organizations and companies from Ethiopia, Northern Nigeria, and one state in India (Uttar Pradesh), so we do not know quantitatively if MNH indicators have been monitored and if they showed any improvement. Despite the lack of objective results of how well MNH services are delivered, the findings are helpful to establish the need for dialog and collaboration among all parties. The application of the principles of effective aid is either positively enabling or negatively undermining the adoption and scale-up of initially donor-funded MNH interventions in these three settings. The findings are very important and indicate clear gaps in the way aid is provided, such as lack of investment in strengthening health systems which limits the scalability of MNH interventions.

The article also describes the role of each of the three actors in “hitting the bull’s eye”: recipient government, donor and technical implementer have to ensure the successful scale-up of MNH interventions. The findings imply an evident lack of dialog and structures for joint and effective planning and coordination to scale up MNH interventions. The article confirms the weak role of the recipient government in negotiating what aid is delivered and how. If the Ministry of Health (MoH) is the rightful owner of the MNH improvements, in accordance to the ownership principle, it is essential that it lead and coordinate scale up. The government of a country is responsible for ensuring the right to health of all its citizens^3^ and therefore must develop the capacity to effectively coordinate and negotiate aligned, harmonized, transparent and predictable aid from their donors. As described by the authors, aid continues to “perennially” be donor-driven and delivered through vertical and parallel structures that are not aligned with the country’s health development priorities and health system organization and functions and which cannot and are not sustained after the a donor-funded project ends. This perennially ineffective and unsustainable way of delivering aid was reported to also negatively impact on communities who not only stop receiving the services they grew accustomed to and are disillusioned by the interruption when aid ends, but who are likely to experience negative health outcomes because of the interruption in healthcare after the donor-funded project ends. The ultimate impact on the “beneficiaries” of this traditional way to of delivering aid was not part of this study, though.

We agree with the authors that “hitting the bull’s eye” is achieved by actually following the six aid effectiveness principles to delivery MNH services at national scale. If actually adopted, the principles studied in this article and other principles such as managing by results and coordinating with multi-sectoral development programs will help address systemic gaps such as lack of healthcare providers, medicines, equipment and facility maintenance and other health-related issues such as gender, water and sanitation, and nutrition. The evidence from the article shows how these principles have become essential to define what aid must be delivered, where, when and how.

To “hit the bull’s eye,” how aid is delivered must change. Aid must include the scale up of the donor-funded and developed interventions by the country’s health system. Scale-up, not just the plan for it, must be part of the scope of work of donor-funded projects. Otherwise, low- and middle-income countries (LMICs) are left to their resources to figure out what to do and how to scale up the new interventions of which they were not part of and with limited information and support. It is not surprising that the country’s health system which should have been strengthened through effective aid is not able to adopt and scale up MNH interventions and MNH indicators continue to be poor. In the light of poor MNH outcomes, the donor then creates a new follow-on project to do it again and the cycle of dependence continues. This article provides additional evidence for governments, donors and implementors in at least in these three countries to come together and change how aid is delivered by following the six principles.

How can governments, donors and implementors apply the six principles and ensure they “hit the bull’s eye?” Effective communication and accountability of all involved seems to be first step to ensure country ownership. Health systems in LMICs differ in degree of performance and organization as it can be seen in the three country examples in this paper. However, they have some characteristics in common that need to emphasized to effectively apply the six principles. They all have an MoH that is the owner and is in charge of country’s leadership in the health sector. The MoH must have a donor coordination strategy implemented across all its programs because it is responsible for ensuring the right to the highest attainable standard of health^3^ of all the country’s citizens (most countries are signatories of the right to health international treaties and conventions) and they are also responsible for managing the majority of the health facilities. The organization and management structures and procedures of MoHs need to be strengthened to ensure their leadership and capacity to manage donors and implementers that execute donor-funded projects and the scale up from pilot to nationwide scale. In most LMICs, the MoH is the main employer in the health sector, followed by faith-based organizations, CSOs and the private sector, formally accredited or not, in various proportions. How each of health worker performs to meet MNH quality standards can be scaled from the MoH platform if it is strengthened to adopt the new interventions.^4^

Effective aid is by design not by default.^5^ MoHs must be able to work with donors side-by-side so donor-funded projects are jointly designed, implemented and monitored. In this way, donors will be actually able to strengthen and improve the performance of the country’s health system, its programs and service delivery facilities. And the way to deliver effective aid by following the six principles and aligning the pilot projects with the country’s systems. As shown by this article, most projects are not designed to be aligned with the country’s health development plan, policies, programs and services or to be scaled up. This is further complicated and made more inefficient when numerous donors work in the same field such as HIV/AIDS through unaligned and unharmonized projects. In sum, projects must not only deliver MNH services but also improve how the country’s MNH programs work and how the health system delivers MNH services so the country can continue doing what the project started. Yes, it is a different way of working in health development that requires all of us to change how we work.

It is important to note the role of the implementer must go beyond project implementation. The implementer has the technical know-how the country needs and must also work as an honest broker between recipient country and donor, translating their respective agendas and timelines and assisting to ensure fluent communication and coordination and well as modeling transparency and accounting for progress towards sustainable project results. There are not many models of effective coordination at this time yet, except the Country Coordinating Mechanism (CCM) of the Global Fund. Most of the time implementers are limited by “project deliverables” and achieving their objectives, which were defined when the project was designed, sometimes one or two years before the project actually started because of the time donor bidding and implementer selection processes take. When grants are involved, implementors have a bit more flexibility to respond to changes in opportunities and priorities, but when contracts are involved, country governments, implementers and donors need to work hard to get a change in “Scope of Work.” A more embedded approach within the health system might help address the need for harmonization, responsiveness and flexibility when improving the performance of health systems in LMICs.

The paper could have been strengthened with clear and actionable recommendations to improve aid effectiveness by applying the six principles for each of the three countries. We hope this is the start of more research in the topic of aid effectiveness, including quantitative and qualitative measurement of actually effective and sustainable country-led programs that strengthen recipient country’s health system. The quantitative indicators of the Paris Declaration and further quantitative research using measures such as the Global Partnership for Effective Development Co-operation (GPEDC) framework^6^ would allow government to compare the effectiveness of various interventions and approaches will be required in the future. Furthermore, study of the effectiveness of other non-project aid such as Global Fund grants and their country coordinating mechanisms also deserve to considered. The authors should focus their research in these three countries as “sentinel” countries for monitoring aid effectiveness. Without further research, there is little information on the actual impact of aid because recipient country’s health information systems and the donor-funded project’s monitoring do not measure trends of maternal and newborn mortality in project sites during and after a project ends. There is need to create new ways to measure effectiveness.^7^ The authors should continue their research in other programmatic topics such as disease control programs, epidemic control and health services coverage expansion.

In conclusion, business as usual does not deliver lasting results at scale. To achieve Sustainable Development Goal 3 (SDG3) targets, recipient countries need to own their health development plan, policies and programs, take charge, and lead an active role to benefit from donor aid and create mechanisms that help them do that. Donors need to change how they plan and deliver aid in a planned and predictable way that measurably strengthens the countries’ health programs (eg, MNH, HIV/AIDs, malaria, tuberculosis [TB], non-communicable diseases, etc) and system so they can continue delivering improved healthcare before and after the projects end at least to a certain previously determined degree. Implementers need to also change from “doing it themselves” to creating new tools and approaches to help implement the country’s development plan to achieve SDG3 and policies. It is not about business as usual, about positioning for the follow-on project and protecting their institutional know-how but effective aid is about “hitting the bull’s eye” and supporting the MoH to take ownership. Implementers must work through the country’s health programs and strengthen the organization and performance of the country’s service delivery facilities and health workforce. They should not set up separate management structure but work through the MoH and its district health offices. In short, implementers need to put themselves in the shoes of the MoH’s Director of MNH, HIV/AIDS, Malaria or TB program and help him or her succeed. Yes, the goal is to get ourselves of the MNH job so we can move on to assist in other health topics such as expanding access to surgical care, trauma, cancer and diabetes, to name a few.

## Ethical issues

Not applicable.

## Competing interests

Author declares that she has no competing interests.

## Author’s contribution

EB is the single author of the paper.
